# Embodied language of emotions: Predicting human intuitions with linguistic distributions in blind and sighted individuals

**DOI:** 10.1016/j.heliyon.2023.e17864

**Published:** 2023-06-30

**Authors:** Michelle Giraud, Marco Marelli, Elena Nava

**Affiliations:** Department of Psychology, University of Milano-Bicocca, Piazza dell’Ateneo Nuovo 1, 20126, Milano, Italy

**Keywords:** Body, Emotion, Language, Blindness, Distributional semantic model

## Abstract

Recent constructionist theories have suggested that language and sensory experience play a crucial role not only in how individuals categorise emotions but also in how they experience and shape them, helping to acquire abstract concepts that are used to make sense of bodily perceptions associated with specific emotions.

Here, we aimed to investigate the role of sensory experience in conceptualising bodily felt emotions by asking 126 Italian blind participants to freely recall in which part of the body they commonly feel specific emotions (N = 15). Participants varied concerning visual experience in terms of blindness onset (i.e., congenital vs late) and degree of visual experience (i.e., total vs partial sensory loss). Using an Italian semantic model to estimate to what extent discrete emotions are associated with body parts in language experience, we found that all participants' reports correlated with the model predictions. Interestingly, blind – and especially congenitally blind - participants’ responses were more strongly correlated with the model, suggesting that language might be one of the possible compensative mechanisms for the lack of visual feedback in constructing bodily felt emotions.

Our findings present theoretical implications for the study of emotions, as well as potential real-world applications for blind individuals, by revealing, on the one hand, that vision plays an essential role in the construction of felt emotions and the way we talk about our related bodily (emotional) experiences. On the other hand, evidence that blind individuals rely more strongly on linguistic cues suggests that vision is a strong cue to acquire emotional information from the surrounding world, influencing how we experience emotions.

While our findings do not suggest that blind individuals experience emotions in an atypical and dysfunctional way, they nonetheless support the view that promoting the use of non-visual emotional signs and body language since early on might help the blind child to develop a good emotional awareness as well as good emotion regulation abilities.

## 1 Introduction

### 1.1 The role of language in the construction of emotions

In everyday life, people commonly use language as a vehicle to communicate their thoughts, feelings, mental states and, more generally, to interact with others. Language is a fundamental tool that permeates every aspect of people's lives, but, despite this, common sense suggests that language has little to do with emotions. Many psychological theories of emotion converge in stating that language mainly serves to express feelings and that emotions are physical types, domain-specific and innate, essentially distinct from linguistic or conceptual processing [1,2,3] and produced within specific neural signatures (see Ref. [4] for a review).

However, more recent psychological research suggests that language goes beyond the simple purpose of describing and communicating an emotion but is constitutive of emotional experiences and perception. According to constructionist theories, especially the Conceptual Act Theory (CAT) [4,5,6,7] and the Theory of Constructed emotion (CET) [8], language scaffolds concept knowledge in humans, helping them in the acquisition of abstract concepts such as emotion categories across development [9]. Children become able to perceive emotion on faces as they learn the meanings of emotion words and start to use emotion words to describe and communicate affective sensations in their bodies and emotional expressions seen in others’ bodies. Before the acquisition of language, infants have basic pleasant, unpleasant, and neutral affect notions (i.e., emotional valence) that gradually narrow into more specific emotion concepts (e.g., anger, happiness, sadness, etc.) throughout the two first decades of life [10,11]. In fact, as toddlers begin to learn emotions words, they start to construct perception of discrete emotions from faces [12] and learn to categorise emotions in the same way they learn about other abstract conceptual categories: by hearing caregivers labelling and providing visual feedback to different events [13,14].

Later on, language aids adults in gaining and using concept knowledge to make sense of continuous and “online” sensory perceptions, connecting concepts to embodied experiences and directing the ongoing processing of sensory input from the body and world to generate emotional experiences and perceptions [9].

People's ability to perceive emotion in faces is hampered when they lack access to the meaning of emotion words, as observed in the study by Gendron and colleagues [14], which temporarily reduces the accessibility of an emotionally relevant word (e.g., anger), impaired the perceptual priming of emotional faces (e.g., frowning eyebrows), thus showing that the same angry face can be encoded differently according to the accessibility to the corresponding word category. Anticipating CAT and CET by three decades, Schachter and Singer (1962) also had the same insight and pointed to a linguistic component for emotional processing, describing the emergence of feelings as follows: “given a state of physiological arousal [ …], the individual will *label* his state and describe his feelings in terms of the cognitions available to him.” [15].

### 1.2 Transforming bodily feelings into conceptual knowledge of emotions

Three fundamental elements contribute to the feeling of emotions: representations of sensations from within the body (known as interoceptive sensations), representations of sensations from outside the body (known as exteroceptive sensations, e.g., sensations conveyed by the sensory organs), and concept knowledge used to make those sensations meaningful in the context [12,16,17]. Experiencing an emotion, such as *fear*, occurs when information from one's own or other people's bodies (e.g. a beating heart, sweaty palms, and shivers down the spine) and exteroceptive sensations (e.g., the sights and sounds of a person following us) is made meaningful in light of the current situation (e.g., being in a dark alley) through the use of conceptual knowledge about emotion (i.e., situated conceptualisation). These concepts represent the knowledge about the single, discrete emotion category, which in turn is learned through language, social interaction, and personal experience [6].

In this constructionist approach, the conceptual knowledge at the core of emotional processing involves a series of embodied experiences. Indeed, all mental states are embodied conceptualisations of internal bodily sensations and incoming sensory input [7], in which the emotional output results from linguistic and motor behaviours [7]. Emotions are often felt directly in the body, and somatosensory feedback helps us to channel these bodily sensations into a conscious emotional experience. This link between emotion and bodily states has also been demonstrated in a series of behavioural studies showing that emotions seem to be related to specific body parts (e.g., anger is mostly located in the hands; happiness is mostly located in the upper body, as the chest), irrespective of cultural background [18,19,20]. This bond between body and emotion also shows a bidirectional relationship, by which a change in bodily signals (e.g., false feedback of increased heartbeat, an increase of cholecystokinin receptor, or a manipulation of facial expression and posture) trigger a change in emotion perception (e.g., intensity/salience faces increases; increase the perception of negative emotion, such as anxiety; and perceive faces as happier, etc.) [21,22,23,24,25]. This relationship also seems to be reflected in how we talk about emotion. When we talk about our feelings, we commonly use a specific emotion language-based that refers both to bodily states (“*Boiling with anger*”; “*Have cold feet*”, etc.) perceived during the emergence of emotion and to more direct sensory experiences, such as vision (“*See how I feel’*"). The use of these metaphoric expressions, such as “*Feeling butterflies in the stomach”* or “*Shiver down the spine*”, suggests that emotions are rooted in bodily states, which, in turn, might have paved the way for the construction of emotional concepts.

This multidimensional characterisation has also been corroborated by neuroimaging studies documenting that emotions are neurally represented as a set of semantic features in distributed motor, somatosensory, linguistic, and affective networks [26]. For example, Damasio and colleagues [27] demonstrated that feeling emotions requires the engagement of brain regions involved in the homeostasis of internal body states, such as the somatosensory cortices and upper brainstem nuclei. The temporary disruption, through repetitive transcranial magnetic stimulation (rTMS), of the face region in the right somatosensory cortex (rSC) and the right occipital face area (rOFA) impaired the discrimination of facial emotion expression but not the ability to identify the face [28]. Moreover, brain regions commonly implicated in language function (e.g., the anterior temporal lobe and ventrolateral prefrontal cortex) also show increased activity during emotion perception; in contrast, individuals with semantic deficits show impairment in discrete emotion perception. In a recent study, Linquist and colleagues (2014) have shown that patients with semantic dementia, a progressive neurodegenerative disease characterised by significant impairments in concept knowledge availability and use, spontaneously perceive the valence of facial expressions (e.g., pleasantness and unpleasantness), but not discrete emotions (e.g., anger, disgust, fear, or sadness), even in a task that does not require the use of emotion words [29].

### 1.3 Seeing emotions: the role of vision in constructing emotions

A great deal of information regarding an individual's emotional state is conveyed by visual social cues, such as facial and bodily expressions [30,31,32], and the development of emotional concepts also comes through seeing the emotional expressions of other individuals. Indeed, while internal subjective states are not visible, they can be represented or transmitted through different channels (e.g., prosody, vocalisations, posture, music, etc.), but primarily through facial expressions. Seeing others' facial movements and further contextual information helps children understand whether they should approach or avoid others, whether their social partner allows their behaviour and whether an environment is secure. In fact, before children's word production acquisition, they use facial movements to guide their actions [33]. The importance of visual interaction has also been emphasised by proponents of dynamic and interactional theories of emotion, who agree that initiating emotion learning processes can be traced back to infant-caregiver eye-to-eye interaction [34]. Specifically, contingent mirroring of parental affect provides infants with social feedback that regulates emotional states and constructs self-representation [35,36]. The classical “still face” paradigm suggests that the perception of one's emotional states also passes through the online visual observation of the caregiver's behaviours [37]. In this paradigm, an experimenter (i.e., an instructed caregiver) interacts with an infant by engaging in playful behaviour, followed by an emotionally neutral still-face phase, and then returning to playful interaction. The still-face paradigm is often used to study emotion regulation in infants, and it shows how some emotional processes can pass through visual information without any verbal communication.

Considering the constructionist approach (e.g., CAT and CET), visual information, along with other sensory information, is one of the elements contributing to the emergence of emotion concepts. Three elements are essential to learn and achieve emotion concepts (e.g., the concepts of fear): bodily internal states, external sensory information, and conceptual knowledge enriched by prior experiences [38]. Nevertheless, psychological compounds like emotions are more than the sum of these three components [39] and we can hypothesise that a loss or variation in bodily states, exteroceptive sensations or conceptual knowledge could lead to also a cascade effect and show changes in the outcome (e.g., emotion generation and perception).

Visual deprivation could have a negative effect on the conceptualisation of emotions. Although blind people also have other ways to experience the world (e.g., touch and hearing), they cannot learn the relationship between people's emotions and the events that trigger them through the direct visual perception of people's facial expressions [40]. Studies have shown that emotion processing may depend upon typical visual experience, in that blind individuals are able to recognise emotions, but their brain uses different processes to sustain such ability [41,42]. This emotional ‘reorganisation’ in the blind brain is supported by behavioural studies showing that blind individuals do not display impairments in emotion processing [43]. On the contrary, differences can be found in how blind individuals produce facial emotional expressions: for example, even if blind and sighted participants spontaneously produce the same pattern of facial expression, differences can be found in facial and body movements' specificity and intensity and control of emotions in certain contexts. Moreover, blind individuals seem to have difficulty in simulated posed emotional expressions, suggesting that visual experience affects in some way the production of voluntary expression and their control [34].

Even by considering other sensory channels (e.g., hearing and sense of smell) and the internal bodily states component, it appears that blind individuals possess superior abilities when performance relies on exteroceptive information (e.g., discriminating and identifying emotions from body odours or auditory discrimination task [44,45]), and higher accuracy in discriminating interoceptive sensations [46,47]. At the net of their emotion perception abilities, they appear to have higher sensitivity to their inner bodily states and more sensitivity to other sensory channels beyond the visual one. Recent studies have revealed that blind individuals show significantly higher accuracy in perceiving their heartbeat than sighted individuals [46]. Changes in afferent interoceptive inputs from the heart can modulate emotions perception (e.g., enhanced perception of fear [48]), in turn modulating the ascending interoceptive signals in the brain. Moreover, blind individuals show differences in how they perceive affective touch by rating it as more pleasant than sighted controls [47]. Pain is also systematically altered in blind individuals: congenitally blind participants display lower pain threshold to heat, greater sensitivity to cold pain stimuli, and higher ratings of pain experienced in response to suprathreshold laser stimuli [49,50,51,52].

Thus, increased sensitivity to body signals and the lack of an exteroceptive signals (such as sight) may modulate body-brain interactions and lead to changes in emotion processing and how blind individuals use emotion-based language. It could be hypothesised that the construction of the internal/emotional world might be strongly affected by language experience, as these individuals cannot rely on visual feedback in social interactions to shape and refine their conceptual representations of emotions, especially when asked to conceptualise their bodily experiences of emotions.

Here we investigated whether vision can shape bodily representation of emotions by asking blind participants to report in which part of their body they feel discrete emotions and comparing their responses to the predictions of computational models trained on purely linguistic data. In particular, based on a constructionist framework, we predicted that if vision contributes to the perception of our internal states, blind individuals should rely more on language than the sighted. This hypothesis is based on the assumption that emotional knowledge is indeed a visceral and sensory experience, but the way we talk about them might shape its meaning and, in turn, the way we feel them.

The overarching goal of this study is to investigate how the absence of vision and different sensibility for interoceptive signals may influence the construction of emotion concepts by seeing if there are any differences in how participants located emotion in body parts. Altogether, these findings might significantly impact our knowledge of visual experience and body function in emotion processing.

## 2 Materials and methods

### 2.1 Participants

We tested one hundred and twenty-six Italian participants with varying experience with blindness onset (i.e., congenital vs late) and degree of visual experience (i.e., total vs partial sensory loss). We enrolled thirty-one congenital individuals (16 males and 15 females; mean age ± SD, 37,9 ± 13,5); thirty-two late individuals (18 male and 14 females; mean age ± SD, 46, ± 12,5); and 32 visual impaired individuals (15 male and 17 females; mean age ± SD, 45,3, ± 16,5). In addition, thirty-one sighted participants (16 males and 15 females; mean age ± SD, 41,6, ± 16,6) were chosen as a control group and matched to participants with visual deprivation by age, gender and educational level (see, Table 1). The sample size was chosen following G*Power a priori sample size calculation (correlation, two-tailed, 0.25 effect size, α err. prob. 0.05 and power 0.8, N = 120).

**Table 1:** Blind participants characteristics. The participant ID code is divided into the three groups of participants with different degrees of visual experience: V stands for Visually Impaired; L stands for Late; and C stands for Congenital blind.

Participants	Age (years)	Sex	Cause of blindness (etiology)	Onset blindness in years	Visual residual (at time of testing)	Handedness
VI04	M	77	Degenerative retinopathy	21	Lateral vision	right-handed
VI05	M	24	Congenital glaucoma	2	Partial Vision (1/10 residual)	right-handed
VI06	F	31	Cerebellum tumour with optic nerve injury	5	Vision from left eye only with central field of view	right-handed
VI07	F	54	Retinitis pigmentosa	15.5	Partial Vision (2/10 residual)	right-handed
VI08	M	25	Retinitis pigmentosa	11.5	Partial vision	ambidextrous
VI09	F	30	Retinitis pigmentosa	2.5	Partial Vision (5/10 residual)	right-handed
VI10	F	41	Cataract	30	Light, shadow and contrast colour	left-handed
VI11	F	46	Retinitis pigmentosa	16	Partial Vision (3/10 residual)	right-handed
VI12	M	54	Congenital glaucoma	18	Light, shadow and contrast colour	right-handed
VI13	F	39	Neonatal asphyxia	36	Partial vision	left-handed
VI14	M	53	Retinitis pigmentosa	2.5	Light, shadow and contrast colour	right-handed
VI15	M	49	Retinitis pigmentosa	2.5	Light, shadow and contrast colour	right-handed
VI16	M	42	Retinitis pigmentosa	9.5	Light, shadow and contrast colour	right-handed
VI17	M	35	Retinitis pigmentosa	14	Light, shadow and contrast colour	right-handed
VI18	M	34	Optic nerve damage	12	Light, shadow and contrast colour	right-handed
VI19	F	47	Congenital glaucoma	2.5	Partial vision	right-handed
VI20	M	29	Bilateral optic nerve atrophy	9	Partial Vision (1/10 left, 0.5/10 right residual)	left-handed
VI21	F	20	Septo-optic dysplasia	27	Light, shadow and contrast colour	right-handed
VI22	F	49	Retinitis pigmentosa	18	Light, shadow and contrast colour	right-handed
VI23	M	60	Retinitis pigmentosa	36	Light, shadow and contrast colour	right-handed
VI24	F	71	Cataract, Glaucoma and maculopathy	46	Vision from left eye only with central field of view	right-handed
VI25	F	34	Rheumatoid Arthritis	25	Light, shadow and contrast colour	right-handed
VI26	F	22	Congenital glaucoma	6	Partial vision	right-handed
VI27	M	36	Unknown	2.5	Partial vision	right-handed
VI28	M	57	Unknown	4	Partial vision	right-handed
VI29	F	44	Stargardt syndrome	6	Partial vision	right-handed
VI30	M	43	Usher syndrome	16	Partial vision	right-handed
VI31	F	60	Degenerative retinopathy	6	Partial vision	right-handed
VI32	M	73	Retinitis pigmentosa	6.5	Partial vision	right-handed
VI33	F	55	Left eye agenesis	13	Partial vision	right-handed
VI34	F	85	Glaucoma	4	Partial vision	right-handed
VI35	F	30	Retinitis pigmentosa	13.5	Partial vision	right-handed
L01	F	33	Bilateral Retinoblastoma	2	No vision	right-handed
L02	F	44	Retinoblastoma	3	No vision	right-handed
L03	M	20	Unknown	5	No vision	right-handed
L04	F	33	Unknown	6	No vision	right-handed
L05	F	48	Unknown	6	No vision	right-handed
L06	M	39	Leukaemia	6	No vision	right-handed
L07	M	21	Congenital glaucoma	6	No vision	ambidextrous
L08	F	53	Retinitis pigmentosa	6	No vision	left-handed
L09	F	30	Neurofibromatosis	7	No vision	right-handed
L10	M	24	Unknown	7	No vision	right-handed
L11	F	55	Rheumatoid arthritis	8	No vision	right-handed
L12	M	42	Glaucoma	8	No vision	right-handed
L14	M	44	Congenital glaucoma	9	No vision	ambidextrous
L15	M	48	Usher syndrome	12	No vision	right-handed
L16	M	52	Optic nerve atrophy	14	No vision	right-handed
L17	F	53	Congenital glaucoma	15	No vision	right-handed
L18	M	51	Retinitis pigmentosa	15	No vision	right-handed
L19	F	65	Preterm birth	17	No vision	right-handed
L20	F	48	Tapeto-retinal degeneration	18	No vision	right-handed
L21	M	43	Unknown	19	No vision	right-handed
L22	F	40	Retinitis pigmentosa	20	No vision	left-handed
L23	M	44	Congenital Leber Amaurosis	20	No vision	right-handed
L24	F	51	Unknown	20	No vision	ambidextrous
L25	F	37	Congenital glaucoma	22	No vision	right-handed
L26	M	52	Congenital Leber Amaurosis	26	No vision	right-handed
L27	M	76	Traumatic event	27	No vision	right-handed
L28	M	51	Traumatic event	28	No vision	right-handed
L29	M	69	Retinitis pigmentosa	28.5	No vision	right-handed
L30	M	51	Retinitis pigmentosa	30	No vision	left-handed
L31	F	52	Retinitis pigmentosa	30	No vision	right-handed
L32	M	52	Traumatic event	30	No vision	right-handed
C01	M	39	Traumatic event	At birth	No vision	right-handed
C02	F	28	Congenital glaucoma and corneal dystrophy	At birth	No vision	right-handed
C03	M	54	Optic nerve atrophy	At birth	No vision	ambidextrous
C04	M	21	Retinopathy of Premature (RoP)	At birth	No vision	ambidextrous
C05	F	20	Unknown	At birth	No vision	right-handed
C06	M	36	Optic Nerve Hypoplasia	At birth	No vision	right-handed
C07	M	40	Retinopathy of Premature (RoP)	At birth	No vision	ambidextrous
C08	F	23	Retinitis pigmentosa	At birth	No vision	ambidextrous
C09	M	23	Retinitis pigmentosa	At birth	No vision	right-handed
C10	M	63	Retinitis pigmentosa	At birth	No vision	right-handed
C11	F	34	Retinopathy of Premature (RoP)	At birth	No vision	right-handed
C12	F	31	Microphthalmia	At birth	No vision	right-handed
C13	F	49	Optic nerve atrophy caused by rubella	At birth	No vision	left-handed
C14	M	49	Traumatic event	At birth	No vision	right-handed
C15	F	40	Retro-ventricular fibroplasia	At birth	No vision	right-handed
C16	F	55	Retinitis pigmentosa	At birth	No vision	right-handed
C17	M	39	Glaucoma	At birth	No vision	left-handed
C18	M	64	Traumatic event	At birth	No vision	right-handed
C19	M	21	Unknown	At birth	No vision	right-handed
C20	F	24	Retinitis pigmentosa	At birth	No vision	right-handed
C21	M	26	Problems during pregnancy	At birth	No vision	right-handed
C22	M	22	Retinopathy of Premature (RoP)	At birth	No vision	right-handed
C23	M	43	Retinitis pigmentosa	At birth	No vision	left-handed
C24	F	40	Retinitis pigmentosa	At birth	No vision	right-handed
C25	M	39	Retinitis pigmentosa	At birth	No vision	right-handed
C26	F	27	Retinitis pigmentosa	At birth	No vision	right-handed
C27	F	22	Retinitis pigmentosa	At birth	No vision	right-handed
C28	F	47	Unknown	At birth	No vision	right-handed
C29	F	61	Traumatic event	At birth	No vision	left-handed
C30	F	52	Tumour	At birth	No vision	right-handed
C31	F	43	Congenital cataract	At birth	No vision	right-handed

The study was approved by the Ethical Committee of the University of Milano-Bicocca (Protocol Number: 610). All participants took part in the study voluntarily and gave their informed consent before participating. Detailed tables reporting response frequency matrices for emotion-body associations, and correlations of emotion-body associations with the distributional semantic model, Word-Embeddings Italian Semantic Space (WEISS-2), can be found at the following link: https://osf.io/7gx9z/.

### 2.2 Stimuli & procedure

All the participants were interviewed over the phone and asked in which body part they felt fifteen discrete emotions through a semi-guided interview. The emotions sampled were the same as those used in the study by Nummenmaa and colleagues (2014), who found interesting new results that shed light on the relationship between emotions and the body and how emotions are embodied [18,19,20]. Detailed instructions were given to each participant at the beginning of the interview and repeated as needed. The researcher listed, one at a time, the emotions to the participant (N = 15), and for each emotion, they were asked to try to indicate in which part of the body they felt physical sensations associated with that discrete emotion, also trying to recall past emotional episodes related to that emotion to help recover the description of body sensations and be as accurate as possible (e.g., “*Now I ask you to focus and think about the emotion of “anger.” Take a few minutes to think about how you feel when angry. You can also help yourself by thinking about prior experiences. Now that you have this emotion in mind and how it makes you feel, can you indicate in which parts of your body you feel it?“)*. At the end of data collection, 14 body districts emerged in common with the responses of all participants; each body district was assigned +1 point when the participant indicated that body part as significantly associated with the emotion they were asked to recall. Zero was given to those body districts that were never mentioned by participants as being related to the given emotion. For example, if for the feeling “love”, a participant answered “head,” “chest,” and “hands,” +1 was assigned to each of these body districts and so on (see, Table 2). In the end, we created a separate matrix, formed by 15 emotions and 14 body districts, for the each of the four groups of participants (i.e., congenitally blind, late, visually impaired and sighted individuals).

**Table 2:** Emotions categories used in the semi-guided interview and the Body districts emerged in common with the responses of all participants.

Emotions categories	Body district
Anger	Head
Love	Face
Depression	Neck
Sadness	Shoulder
Anxiety	Arm
Envy	Hands
Pride	Trunk
Disgust	Belly/Stomach
Sham	Back
Surprise	Gluteus
Happiness	Genitals
Contempt	Legs
Neutral (Calm)	Feet
Fear	Whole Body
Serenity	

### 2.3 Statistical analysis

In order to estimate the degree of association between emotions and body parts in language experience, we used a distributional semantic model, which allows us to obtain semantic estimates for a large number of words in an intuitive and fast way. The principle behind this approach is the distributional hypothesis [53]: the meaning of a word is (or can be approximated by) the contexts in which that word appears. From a computational point of view, this means extracting lexical co-occurrences from extensive collections of texts. We decided to use Word-Embeddings Italian Semantic Space (WEISS-2, http://meshugga.ugent.be/snaut-italian-2/) [54], model trained on Italian data, consistently with the language spoken by our participants. This model is released through the SNAUT website [55] and we used to extract the cosine proximity for all possible pairs of emotions and body parts as a measure of semantic association. These language-based estimates were first correlated with the emotion-body labels provided by blind and sighted individuals.

In a second analysis, the exact model estimates were used as a predictor in generalised mixed model with a Poisson linking function [56]. This analysis tests the relationship between the variables of interest (i.e., the production patterns in the participants' responses and the model estimates based on language usage) by also accounting for the non-independence of observations via random effects. In this analysis, the dependent variable was represented by the frequency of production, and the language-based estimates represented the independent variables, the group (with the sighted taken as reference level), and their mutual interaction; random intercepts for body parts and emotions were included. This approach allows evaluating the relationship between language-based associations and participants’ productions by considering the stimuli-related structure of the dataset, and in particular the portion of the variance in the responses that could be explained by the repetition of body parts and emotions across different datapoints. Following the estimation of the model, this was refitted by removing datapoints with residuals exceeding 2.5 SD in their distribution, to ensure that the results were not overly influenced by extreme observations.

## 3 Results

**Fig. 1:**
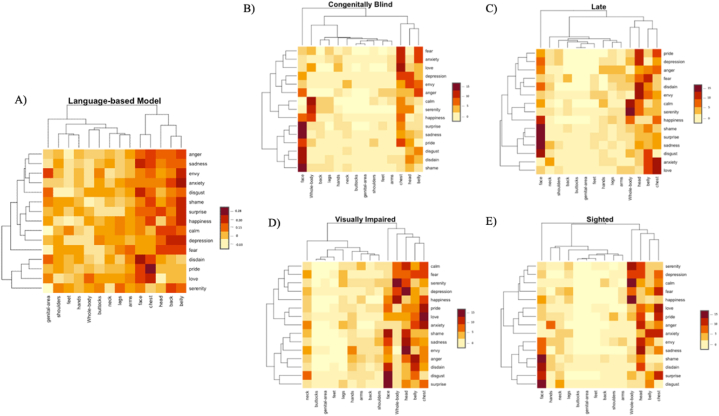
Cluster Heatmaps depicting the distribution of emotion-body part association of the four groups of participants and the language-based model WEISS-2. A) Cluster Heatmaps of the language-based Model, i.e., WEISS-2. B) Cluster Heatmaps of Congenitally blind participants. C) Cluster Heatmaps of Late participants. D) Cluster Heatmaps of Visually impaired participants. E) Cluster Heatmaps of Sighted participants.

Correlations between all the four matrices were positive (all r > 0.79), showing that emotion-body associations are mainly similar, irrespective of visual experience (see Fig. 1). Then, we used the Italian distributional semantic model [57], to measure what extent each emotion label is related to bodily-related words in linguistic data. Correlations between participants' intuitions and model estimates indicated, for all four groups, a significant positive association with the language-based estimates from the distributional model: sighted participants (r = 0.29), congenitally blind (r = 0.38), late blind (r = 0.37) and visually impaired (r = 0.40). These results show that, to a certain extent, all participants align to linguistic associations when asked to provide emotion-body associations. Interestingly, a series of William's tests conducted on the correlations between the model estimates and responses of each of the four groups revealed that blind individuals relied more on the model than the sighted (sighted vs congenitally blind participants: t = −2.22, p < 0.028; sighted vs visual impairment participants: t = −2.9, p < 0.0041; sighted vs late participants: t = −2.04, p < 0.043). We did not find any effect of visual experience nor visual acuity, as no significant difference emerged between blind participants (congenitally blind vs late participants: t = 0.09, p < 0.93; congenitally blind vs visual impairment participants: t = −0.54, p < 0.59; late vs visual impairment participants: t = −0.66, p < 0.51).

The generalised mixed model replicated one of the main findings of the correlation analysis, namely that in all groups, participants' productions were aligned with the degree of association estimated by the language-based model (as assessed by a simple effect of the model estimates p = 0.002). However, this analysis also showed an interaction between group and language-based estimates, with a particularly positive significant parameter for the congenitally blind group (p = 0.003). This indicates that the congenitally blind's productions are more aligned with estimates from language usage as compared to the sighted group. In other words, associations between emotions and body parts in congenitally blind individuals are predicted to a higher degree by patterns observable in language usage when compared to the production of sighted participants. On the contrary, this was not the case for late (p = 0.08) and visually impaired individuals (p = 0.95) (see, Table 3).

**Table 3:** Results of the generalised mixed-effects model predicting participants’ production from language-based semantic associations.

Predictor	Estimate Std.	Error z	Value	Pr (>|z|)
Intercept	0.32168	0.34961	0.920	0.35750
Group: Congenitally blind	−0.52805	0.12245	−4.312	1.62e-05
Group: Visually Impaired	0.20626	0.10328	1.997	0.04581
Group: Late	−0.30501	0.11651	−2.618	0.00885
**Cos**	**1.61337**	**0.51844**	**3.112**	**0.00186**
Group: Congenitally blind * Cos	**2.13912**	**0.71503**	**2.992**	**0.00277**
Group: Visually Impaired * Cos	0.03629	0.63230	0.057	0.95423
Group: Late*cos	1.20980	0.69686	1.736	0.08255

## 4 Discussion

The results from the two analyses revealed important findings, the first of which showed that interoceptive bodily sensations associated with emotions are partially grounded in linguistic experience and thus that language helps shape and scaffold emotions' experience and perception by giving meaning to sensations inside and outside the body within conceptual knowledge [5,6,7,8,12,58]. The semantic distribution model revealed that all participants, irrespective of visual experience, align to linguistic, emotion-body associations (Fig. 1).

These findings appear in line with the Conceptual Act Theory (CAT) [39], which claims that language plays a crucial role in feeling emotions because it supports the conceptual knowledge used to make meaning of the body and world sensations in a given context, helping humans to learn abstract concepts across the lifespan.

However, the models also revealed two important findings related to the specific role of visual experience in shaping the way body-emotion associations are linguistically conveyed. The tests conducted on the correlations between the model estimates and the responses of each of the four groups revealed that all blind individuals, irrespective of visual experience, were more closely aligned with the model than the sighted.

This result suggests that language might provide blind individuals with information not only about conceptual categories more involved in visual experiences, such as colour-adjective associations [59,60] or the physical appearance of animals [61] but also about more abstract and personal concepts, such as emotions. Presumably, the set of subjective experiences of own bodily states (e.g., feeling one's heartbeat or body temperature rise) and linguistic aspects of emotions, such as the use of similes or metaphorical expressions heard in a specific social environment (e.g., feeling heartbroken or chip on your shoulder), allow blind people to develop the same emotion-body associations as in sighted, who can also rely on visual feedback during social interactions to regulate their own internal emotional states and understand others. Therefore, this important finding suggests that emotional experiences are firmly rooted not only in the body and the visual experience we have of them, but also in language.

The analysis relying on generalised mixed models revealed that, more specifically, congenital absence of vision makes the intuitions of blind individuals more aligned with estimates from language usage, as compared to the sighted.

The two analyses are not entirely consistent with each other, in that one shows that the lack of any type of visual experience promotes more adherence to language usage. In contrast, the other one suggests that it is the lack of visual deprivation at birth that determines a higher reliance on language in producing body-emotion associations. This could stem from the more sophisticated second analysis, which takes into consideration the complex structure of our data and account for the non-independence of observations driven by the bodypart-emotion pairs. Nonetheless, it is important to note that both analyses convey the same take-home message that not only language, but visual information play a crucial role in shaping the conceptual knowledge of individuals’ emotional, bodily experiences.

The fact that the second analysis brought up the role of age of blindness as a key factor in shaping linguistic body-emotion associations is in line with studies showing that there is a sensitive period in the responsiveness of visual areas (i.e., occipital cortex) to language. In other words, lack of vision from birth (or at least in the very first months of life) promotes massive plastic reorganisation of the brain, in that areas typically tuned to process visual information are “used” to participate in language processing [62]. This, together with studies showing improved semantic and episodic memory [63], enhanced chemosensory sensitivity to negative emotions [44] and, in general more structural plasticity in the congenitally blind brain [64], are in line with the results of the second analysis, which is supportive of a more age-dependent view of compensatory plasticity that occurs within limited periods of early development.

Overall, our results point to the key role of language and body signals in constructing emotional experiences. Thus, the presence of different compensatory strategies that could help overcome the lack of visual feedback (such as reliance on language and the enhancement in interoceptive sensibility) seems to play a key role in the processing of emotion in an individual with sensory deprivation (e.g., blindness).

In conclusion, we showed that language serves as a bridge between emotional concepts and embodied experiences and that vision contributes to shaping felt experiences with the external world. Language seems to structure individual and collective experiences, allowing emotions to be shared through culturally spread concepts (e.g., labels or generic statements) [57].

Finally, we would like to highlight some limitations of the study as well as provide future research directions. First, the blind participants tested had varying ages (20–75), and some studies have shown a significant age-dependent reduction in the intensity of bodily-related emotions [65]. Although our blind participants were all age-matched with sighted controls, this high age variability might have influenced the results. Second, our sample was constituted of Italian adults only. Extending the study across cultures and in the younger populations (i.e., children) could inform about the role of culture in constructing concept knowledge related to emotions and developing this construction when complex linguistic skills are still missing, respectively.

While our data are suggestive of the role of language and vision in the construction of emotions, they cannot rule out whether participants really re-enacted the emotions during the experiment, being unable to demonstrate a causal link between vision and emotional processing. Neuro- and/or electrophysiological measures could have assessed whether physiological state changes occurred during the emotional recall.

Future studies could be conducted by considering different sensory deprivations (such as deafness), which could shed light on whether there is a specificity of vision or whether sensory deprivation contributes to this result.

Our results have the potential to inform future studies on the tight relationship between sensory experience, embodied emotions and language, and how the absence of sensory feedback can modify the way emotions are felt in the body and, in turn communicated. Our findings can also contribute to understand how blind individuals conceptualise emotions, in turn promoting language as a tool to teach them to distinguish their own emotions as well as the emotions of others. Indeed, gestures and facial expressions, are commonly displayed without an accompanying sound, and teaching both blind children and their teachers to label such visual features of emotions could be a way to help them gain self-awareness of their own and others’ emotional inner world.

## 5 Conclusions

In this study, we investigated the role of visual experience in constructing bodily felt emotions in blind individuals, and found that all individuals, irrespective of visual experience, rely on language to map emotional experiences to body parts. However, blind and especially congenitally blind individuals appear to rely more strongly on language, revealing that both language and sensory experience shape emotions.

Our results show that bodily experiences and emotions are strongly grounded in language, all three of which contribute to the conceptual knowledge of felt emotions. For blind individuals, in particular, language might represent a possible mechanism to compensate for the sensory loss in the construction of bodily felt emotions.

## Author contribution statement

Michelle Giraud: Performed the experiments; Contributed reagents, materials, analysis tools or data; Wrote the paper.

Marco Marelli: Analyzed and interpreted the data.

Elena Nava: Conceived and designed the experiments; Analyzed and interpreted the data.

## Data availability statement

Data associated with this study has been deposited at https://osf.io/7gx9z/.

## Additional information

No additional information is available for this paper.

## Funding statement

This research did not receive any specific grant from funding agencies in the public, commercial, or not-for-profit sectors.

## Declaration of competing interest

The authors declare that they have no known competing financial interests or personal relationships that could have appeared to influence the work reported in this paper.
